# Responding to climate change and the global land crisis: REDD+, market transformation and low-emissions rural development

**DOI:** 10.1098/rstb.2012.0167

**Published:** 2013-06-05

**Authors:** Daniel C. Nepstad, William Boyd, Claudia M. Stickler, Tathiana Bezerra, Andrea A. Azevedo

**Affiliations:** 1 IPAM International Program, 3180 18th Street, Suite 205, San Francisco, CA 94110, USA; 2 Law School, University of Colorado, 401 UCB, Boulder, CO 80309, USA; 3 IPAM, SHIN CA 5, Bloco J2-Sala 306, Bairro: Lago Norte, Brasília DF 71503-505, Brazil

**Keywords:** food security, commodity roundtables, forest conservation, governance, certification, biofuels

## Abstract

Climate change and rapidly escalating global demand for food, fuel, fibre and feed present seemingly contradictory challenges to humanity. Can greenhouse gas (GHG) emissions from land-use, more than one-fourth of the global total, decline as growth in land-based production accelerates? This review examines the status of two major international initiatives that are designed to address different aspects of this challenge. REDD+ is an emerging policy framework for providing incentives to tropical nations and states that reduce their GHG emissions from deforestation and forest degradation. Market transformation, best represented by agricultural commodity roundtables, seeks to exclude unsustainable farmers from commodity markets through international social and environmental standards for farmers and processors. These global initiatives could potentially become synergistically integrated through (i) a shared approach for measuring and favouring high environmental and social performance of land use across entire jurisdictions and (ii) stronger links with the domestic policies, finance and laws in the jurisdictions where agricultural expansion is moving into forests. To achieve scale, the principles of REDD+ and sustainable farming systems must be embedded in domestic low-emission rural development models capable of garnering support across multiple constituencies. We illustrate this potential with the case of Mato Grosso State in the Brazilian Amazon.

## 1. Introduction

Humanity is facing two major, interconnected global environmental challenges. First, anthropogenic climate change is increasing temperatures, weather extremes and sea level, with large negative impacts predicted and already being felt [1–4]. Second, rapid growth in the demand for land-based production (food, feed, fuel, fibre) is outpacing the growth in supply, creating a rise in commodity prices that is driving civil unrest, hunger and malnutrition [5–7]. We refer to this second challenge as the ‘global land crisis’, because the declining amount of land available for agricultural expansion [8,9] is contributing to the imbalance [5]. These global challenges are the broader context for other major global environmental issues that we are facing, including freshwater scarcity, nitrogen loading, the loss of natural ecosystems and biodiversity, as well as the release of toxins into the environment [10,11].

Anthropogenic climate change and the global land crisis are interconnected at several levels. Climate change is driven largely by the increase in radiative forcing of the atmosphere caused by the rising concentration of heat-trapping (i.e. ‘greenhouse’) gases (GHGs), including carbon dioxide, methane, nitrous oxide and others [3]. More than one-fourth of global GHG emissions are associated with land use, and approximately one-half of these emissions emanate directly from agricultural fields, pasture, livestock and agricultural operations (machinery, transport, fertilizer production). The remainder are caused indirectly through agricultural or livestock expansion into forests and savannahs, especially in tropical regions [12]. In addition to land-use emissions, forests absorb approximately one-fourth of annual global emissions, largely in regrowing forests [13]. This global terrestrial carbon sink may be weakening, as climate-change-related increases in tree mortality and forest dieback become more common [14]. Extreme weather events are also beginning to contribute to crop failures [1,2,4], exacerbating the global imbalance in the growth of demand versus supply of land-based products.

How do we increase the growth of land-based production while levelling off then reducing GHG emissions from land use? And how do we achieve this transition in a way that will also address the related problems of freshwater supply, the loss of native ecosystems and biodiversity, toxicity and nitrogen loading? These questions are particularly daunting in the light of the *declining* trends in agricultural yields in many places in the world [15].

A comprehensive global policy, or set of policies, for addressing the rising competing demands for land and land-based production and the links between land scarcity and climate change does not exist, however, and there are no indications that it will be created anytime soon. In lieu of such a comprehensive framework, solutions must be sought in public policy innovations at all levels of governance to protect and restore tropical forests and to support innovation and increases in agricultural and livestock yields in a manner that reduces GHG emissions from land use. Policy approaches can be reinforced through market transformation to favour sustainable practices, and these market-based initiatives can be strengthened through linkages to policy. Some options for initiating these changes include (i) emerging policy frameworks that are beginning to create incentives and compensation for jurisdictional efforts to reduce emissions from deforestation and forest degradation while enhancing carbon storage in natural and managed ecosystems (‘REDD+’, the acronym for ‘reductions in emissions from deforestation and forest degradation [16]), (ii) market exclusion of producers of land-based commodities (food, fuel, feed and fibre crops) who are converting forests and other natural ecosystems to cropland and engaging in other unsustainable practices through agricultural commodity ‘roundtables’ and (iii) domestic policies and markets for promoting a shift to low-deforestation, high-yield land-use systems. Although each of these three processes have been proceeding largely in isolation from one another, there are important emerging opportunities for combining them into a new rural development model, referred to here as ‘low-emission rural development’ (LED-R).

These nascent efforts aimed at establishing pathways to LED-R in key jurisdictions are taking shape against a backdrop of significant fragmentation in climate policy, with GHG compliance systems and other efforts to establish policy frameworks for low-emissions development emerging across multiple levels of governance [17,18]. Even if the United Nations Framework Convention on Climate Change (UNFCCC) Durban Platform ripens into a new international treaty, it will very likely be built upon a plural, pledge-and-review architecture that translates at best into a series of loosely linked domestic and regional compliance systems supported by robust international monitoring, reporting and verification (MRV) [18]. Splintering of climate policy initiatives thus appears to be a basic fact for the foreseeable future; any effort to respond to the dual crises of climate change and land scarcity will have to work within this world of policy fragmentation [19].

Moreover, because so much of land use and rural development is messy and place-specific, effective responses to these crises will only work if they are fashioned in a manner that comports with local institutions. Several decades of social science research have underscored the fact that rural development is not something that can be ‘managed’ through top-down approaches and global strategies conceived and orchestrated from afar [19–22]. At the same time, place-specific, bottom-up approaches are unlikely to succeed in isolation and will never scale without some coordination from above.

As a result, effective approaches to the dual challenges of climate change and land scarcity will necessarily involve multiple actors (public and private) interacting at multiple scales: what the late Ostrom [23,24] called nested, polycentric forms of governance. The UNFCCC is simply one aspect of this, and while it is no longer driving the climate policy process, it can still play an important role in supporting and coordinating ongoing work at national and subnational levels. This is particularly important in the context of policy frameworks to maintain and restore forests, which will require unprecedented levels of coordination across all levels of governance.

In this opinion paper, we review the status of two major international initiatives—REDD+ and market transformation—that could potentially help to overcome the dual challenges of climate change and the global land crisis. We then identify how these initiatives might be linked together to realize this potential more effectively by influencing and aligning with domestic policies, finance and regulations. We examine the critical lessons provided by each initiative, then propose specific steps by which potential synergies between REDD+, market transformation and domestic policy could be realized within the context of an LED-R model that is already beginning implementation in some states of the Brazilian Amazon, and that could expand to include other major agricultural regions around the world.

## 2. REDD+

### (a) UN and affiliated processes

A key component of a global strategy to mitigate climate change while managing the growing shortfall in land-based production is to create incentive systems for maintaining and restoring natural forests. Various efforts over the past several decades to construct a workable global forest governance regime outside the climate policy context have been marked by repeated failures and false starts, with few notable success stories. Despite widespread recognition that tropical forests have been in ‘crisis’ since the early 1980s [25], the international community has moved from one policy approach to another without any overall effort to forge a coherent, performance-based approach that addresses directly the structural tensions embedded in forest governance and the basic forces driving forest destruction. Best known among these are the debt-for-nature swaps of the early 1980s [26], the Tropical Forestry Action Plan, the International Tropical Timber Organization's efforts to leverage international trade to promote sustainable forest management, the Convention on Biological Diversity and the broader international effort to use protected areas as a means of preserving biodiversity hotpots and the UN Forum on Forests [27–31]. Explanations of the failure of global forest governance have focused on a variety of factors, including the variability in the forces driving deforestation, deep-seated conflicts over sovereignty and control of forest resources, and limited institutional and forest governance capacities at national and subnational levels [28,32,33].

Although tropical deforestation was excluded from the Kyoto Protocol (KP), since 2005 there has been a concerted effort within the UNFCCC to develop a climate policy approach to deforestation that would compensate tropical nations which reduce carbon emissions from tropical deforestation and forest degradation [16,34]. Known as REDD+, this effort has emerged as one of the most advanced components of the current round of climate treaty negotiations within the UNFCCC.

Yet, despite considerable progress over the past several years, implementation of an international REDD+ mechanism has been postponed because of delays in the UNFCCC negotiations. A binding agreement within the UNFCCC that could provide a regulatory framework and unified global mechanism for financing REDD+ and other critical components of a global climate treaty will likely not take effect until 2020 at the earliest. The recent stalemate over REDD+ MRV and finance at COP 18 further reinforces how challenging it will be to move forward quickly under the UN process [35].

Notwithstanding the lack of progress towards a new climate treaty and an international REDD+ mechanism, the UN process has produced important guidance on some of the key elements of REDD+, including social and environmental safeguards [36], emissions reference levels [37] and MRV [38], and continues to provide an important forum for information sharing and policy coordination. At the same time, several multi- and bilateral programmes have also been developed to support the UNFCCC REDD+ mechanism with a focus on engaging and delivering finance to tropical nations that are beginning to develop REDD+ programmes. Originally intended as interim sources of fast-track ‘start-up’ funding to help developing nations prepare for a global REDD+ mechanism, these programmes now comprise the majority of international finance that will be available to support REDD+ programmes through 2015 after which new climate policies in California, Australia and elsewhere *may* provide more robust mechanisms for rewarding emissions reductions from deforestation and forest degradation (table 1). The current REDD+ programmes include the Forest Carbon Partnership Facility, administered by the World Bank, which is completing a phase of supporting ‘readiness’ in 36 nations and preparing to channel more than $400 million in carbon finance to tropical nations. The ‘UN REDD’ programme—administered by the UN Food and Agriculture Organization, UN Environment Programme and the UN Development Programme—is supporting 46 developing nations that are interested in developing REDD+ programmes (http://www.un-redd.org). The ‘REDD+ partnership’ joins together donor nations that made an aggregate commitment of $4.1 billion to ‘interim’ REDD+ finance in a process that includes collaboration with developing nation partners ([40]; table 1).

**Table 1.** REDD+ finance available to tropical nations in 2012–2014 and 2015–2020 in US$ millions. Figures estimated on the basis of existing commitments and estimated requests. If information is not available, entry is marked ‘n.a’.

			2015–2020	
sources		2012–2014	(moderate estimate)	(optimistic estimate)
international community of developed nations through UNFCCC and multi-lateral funds	UN-REDD	2.8^a^	n.a.	n.a.
	FCPF-readiness	208^a^	n.a.	n.a.
	FCPF-carbon fund	218^a^	n.a.	n.a.
	FIP	338^a,b^	n.a.	n.a.
	UNFCCC and/or green climate fund and/or developed countries^c^	n.a.	48 750^d^	112 500^e^
international unilateral funds	UK (ICF)	1099^a,f^	n.a.	n.a.
	Germany (ICI)^g^	196^h^	n.a.	n.a.
	Japan-FSF fund	n.a.	n.a.	n.a.
	Norway (ICFI)^i^	n.a.	n.a.	n.a.
Amazon fund (Brazil)	Amazon fund	264^j^	n.a.	n.a.
private funds/investments	e.g. Athelia climate fund	325^k^	1287^l^	2468^m^
markets/offsets	Australia (market)	0	969^n^	1700^o^
	California (market)	0	493^p^	986^q^
	Korea (market)	0	n.a.	n.a.
	Japan-BOCM	0	783^r^	1567^s^
	Rio de Janeiro (market)	0	n.a.	n.a.
	São Paulo (market)	0	81^t^	162^u^
voluntary market	voluntary market	407^v^	1066^w^	1381^x^
total (US$)		3058	53 429	120 764

^a^Climate funds update. These amounts reflect the total deposited less the amount approved or disbursed. Information obtained from data available on the website http://www.climatefundsupdate.org/ (accessed 8 December 2012).

^b^This also includes some FIP pre-approved operations (i.e. US$70 million to Brazil and US$51 million to others).

^c^The green climate fund (GCF) was created through international negotiations under the auspices of UNFCCC. The GCF was proposed in the Copenhagen Accord, in which developed countries promised to mobilize US$ 100 billion per year, starting in 2020, for climate change mitigation and adaptation. The GCF may be able to mobilize a large portion of the resources for REDD+. However, many doubts linger about the GCF's potential for mobilizing resources, especially since countries are demonstrating a growing interest in using more flexible, less bureaucratic mechanisms (such as bilateral agreements).

^d^This is if the GCF/UNFCCC/developed nations are able to mobilize US$ 10 billion starting in 2013, increasing by US$ 5 billion per year until 2020, when it reaches US$ 45 billion per year. Figure indicates the total collected if 25% can be transferred to REDD+.

^e^This is if the GCF/UNFCCC/developed nations are able to mobilize US$ 30 billion starting in 2013, increasing by R$ 20.73 billion (US$ 10 billion) per year until 2020, when it will meet the goal of US$ 100 billion per year. Figure indicates the total collected if 25% can be used for financing REDD+.

^f^This amount is not exclusively for REDD+.

^g^These figures do not include donations made by the German development agency (GIZ) or the Reconstruction Credit Institute (KfW).

^h^Climate funds update. Information obtained from data available on the website http://www.climatefundsupdate.org/ (accessed 1 August 2012).

^i^Norway has made a strong commitment to investing in REDD+ activities. Besides the money it has committed to Brazil, it has also committed US$ 1 billion to Indonesia, US$ 250 million to Guyana, and US$ 72 million to Tanzania, as well as other commitments being formulated for Mexico and Ethiopia and to support nongovernmental environmental organizations. To avoid double counting, the payments to the Amazon Funds have been excluded here and added under Amazon Funds.

^j^Amazon fund. This represents amounts received and amounts to be received, less the amount related to projects already approved by the Fund.

^k^State and Trends of the Carbon Market. Carbon Finance at the World Bank. This value reflects the total amount the Althelia expects to raise [39].

^l^Based on an estimated annual growth of 20% and additional funds.

^m^Based on an estimated annual growth of 40% and additional funds.

^n^Author's calculations, based on the following data: total demand for international offsets estimated at 350 MtCO_2_ (70 MtCO_2_ annually) [39]; demand for REDD+ estimated at 87.5 MtCO_2_ (17.5 MtCO_2_ annually); price per ton of CO_2_ estimated at US$ 10, with an annual increase of 4%.

^o^Author's calculations, based on the following data: total demand for international offsets estimated at 350 MtCO_2_ (70 MtCO_2_ annually) [39]; demand for REDD+ estimated at 87.5 MtCO_2_ (17.5 MtCO_2_ annually); price per ton of CO_2_ estimated at US$ 15, with an annual increase of 5%.

^p^Assuming the maximum demand in California for REDD+ credits would be approximately 7.3 MtCO_2_e per year starting in 2015, which would total approximately 44 MtCO_2_e through 2020. The minimum price for compensations is estimated at US$ 10, readjusted annually at 5%.

^q^Assuming the maximum demand in California for REDD+ credits would be approximately 14.5 MtCO_2_e per year starting in 2015, which would total approximately 88 MtCO_2_e through 2020 [39]. The minimum price for compensations is estimated at US$ 10, readjusted annually at 5%.

^r^Starting in 2012, Japan may generate a demand for up to 539 MtCO_2_e [39] in order to reach the reduction targets established under the auspices of the UNFCCC (−6% in comparison to 1990). This figure assumes REDD+ compensations can be utilized to meet 12.5% *of the demand* at a price of US$ 10 per tCO_2_.

^s^Based on the same data as above, but assuming a demand of 25% at a price of US$ 10 per tCO_2_.

^t^This assumes 50% of the necessary reductions can be achieved through carbon credits and that REDD+ credits can be used to meet 25% of this demand at a value of R$ 20 per tCO_2_.

^u^This assumes 50% of the necessary reductions can be achieved through carbon credits and that REDD+ credits can be used to meet 50% of this demand at a value of R$ 20 (US$ 10) per tCO_2_.

^v^This is based on (i) the average of the volume negotiated in the three years 2009–2011, with an increase in demand of 3% per year, and (ii) the amount paid in 2011, with an annual readjustment of 3%.

^w^This is based on (i) the average of the volume negotiated in the three years 2009–2011, with an increase in demand of 3% per year, and (ii) the amount paid in 2011, with an annual readjustment of 3%.

^x^This is based on (i) the average of the volume negotiated in the three years 2009–2011, with an increase in demand of 5% per year, and (ii) the amount paid in 2011, with an annual readjustment of 5%.

To date, these UN-affiliated processes have engaged dozens of developing nations in dialogues and preparations for REDD+, with a focus on two of the main performance targets of REDD+: forest carbon emissions and social/environmental co-benefits. The level of engagement of developing nations in these programmes and in REDD+ more generally varies greatly, however [41]. In most cases, participating governments have dedicated little time, staff or political capital to REDD+, a problem that is exacerbated by the lack of a robust funding mechanism for REDD+ and by the sheer burden of accompanying the numerous international dialogues and complex language as well as jargon that have developed around REDD+ and that are inherent in UN treaty negotiations [42]. An important contribution of these programmes, however, has been the engagement of a small group of developing nations that are participating in the REDD+ agenda, and actually taking steps to integrate their REDD+ programmes into their domestic policy frameworks. These interim finance programmes have also forged unprecedented, large-scale ‘pay-for-performance’ agreements with tropical nations, led by Norway. For example, Norway has committed up to $1 billion each in ‘pay-for-performance’ finance to both Brazil and Indonesia in support of these nations' REDD+ programmes (table 1). This overview of the status of REDD+ draws on recently published reviews [16,42–45].

### (b) Initiatives outside the UN process

In addition to these UN-affiliated efforts, there are a number of initiatives taking shape outside the UN process that could provide important pathways and learning opportunities for the development of REDD+ programmes at national and subnational scales. An important contribution of some of these efforts has been the focus on *jurisdictional* approaches to REDD+, designing programmes and institutional frameworks that operate across entire nations, states or provinces. One of the most advanced forums for the development of jurisdictional approaches to REDD+ is the Governors' Climate and Forests Task Force (GCF). Established in 2009, the GCF is a collaboration among 19 states and provinces from Brazil, Indonesia, Mexico, Nigeria, Peru, Spain and the USA that seeks to advance jurisdictional programmes for reducing emissions from deforestation and land use and link these activities with emerging GHG compliance regimes and other pay-for-performance opportunities. More than 20 per cent of the world's tropical forests are in GCF states and provinces, including more than 75 per cent of Brazil's and more than half of Indonesia's. The GCF includes early mover states and provinces that are building comprehensive, jurisdiction-wide approaches to low-emissions development and reducing deforestation as well as the only jurisdiction in the world (California) that is currently considering provisions that would recognize REDD+ as part of its GHG compliance system [42,46].

To date, GCF governments have imposed logging bans, implemented ‘wall-to-wall’ land-use zoning and rural law enforcement programmes. Some GCF states and provinces are in the process of adopting comprehensive state-level REDD+ programmes, and enacting (or implementing) novel legislation to create incentives to protect forests and penalize forest destruction. These subnational governments are trying to increase the economic value of their standing forests, while they are building their economies by growing forest-dependent industries [42].

**Figure 1.**
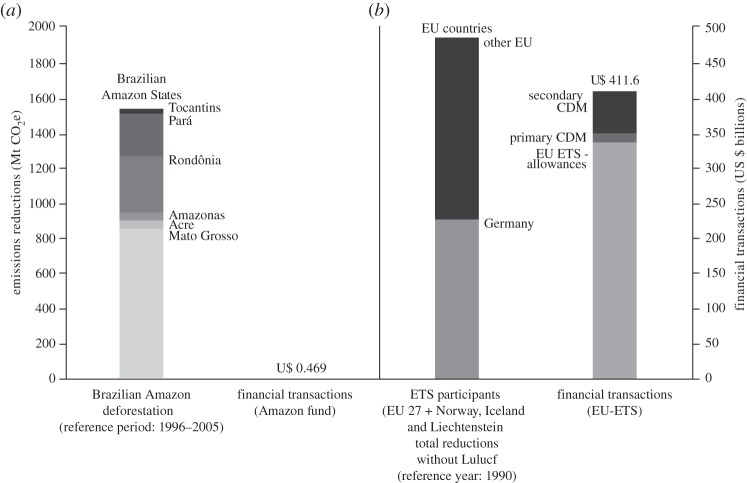
Carbon emissions reductions from states of the Brazilian Amazon (*a*) and European Union signatories to the Kyoto Protocol [39] (KP) (*b*) and associated financial transactions for the first 3 years of the KP compliance period (2008–2010) (Amazon fund. http://www.fundoamazonia.gov.br accessed 20 February 2013) [47].

A recent assessment of REDD+ programme development in the GCF states and provinces found that despite a lack of financial support, several of these states have made considerable progress towards building robust jurisdictional programmes and some have achieved substantial emissions reductions [42]. From 2008 through 2010, the first 3 years of the KP compliance period, the states of the Brazilian Amazon achieved reductions in deforestation with associated reductions of carbon emissions equivalent to 1.5 GtCO_2_, only 0.4 GtCO_2_ less than the European Union, the largest block of nations that are signatories to the KP (figure 1). The EU Emissions Trading Scheme (EU ETS), which is the EU's primary mechanism for complying with the KP, fostered $410 billion in financial transactions (through allowance auctions, offset deals through the Clean Development Mechanism and other mechanisms) during this period. Only $469 million in REDD+ finance was committed to the Brazilian government for reductions in deforestation in the Amazon region—roughly 900 times less than the total financing involved in the EU ETS to date.

Progress across the GCF states and provinces has been uneven and is quite fragile in all cases, threatened by political turnover in many jurisdictions and by the challenges facing governors who must decide if their REDD+ efforts are likely to provide jobs and economic gain sufficient to compensate for the foregone opportunities associated with various deforestation activities. Most GCF states and provinces have yet to realize any financial benefits from their REDD+ efforts, and most of the current international finance dedicated to REDD+ efforts has not directly funded subnational governments. These constraints on the delivery of finance to early mover states and provinces are occurring at a time when state- and province-level activities are emerging as important examples of innovative, ‘bottom-up’ efforts to develop programmes and regulations for jurisdictional approaches to REDD+ and, in some cases, to achieve high-quality, verifiable emissions reductions that could be accepted as offsets in emerging GHG compliance systems such as California's cap-and-trade programme [42].

## 3. Domestic policies and programmes for forests and agriculture

The success of international policy frameworks in fostering the maintenance and restoration of forests and sustainable agricultural intensification depends, in large part, on the degree to which they build upon and facilitate policy alignment, rule of law and cross-sector engagement in tropical developing nations. National and subnational governments control large flows of public spending (e.g. agricultural loan programmes), regulatory frameworks (e.g. land-use laws, forest concession systems), tax structures and law enforcement. One measure of the importance of government policies and programmes is the scale of spending on agriculture. Low- and middle-income nations spend US$160 billion [48] per year on their agricultural sectors (2005–2007), 16 times more than the sum of annual spending into these sectors by Official Development Assistance (US$7 billion [49]) and foreign direct investment (US$3 billion [50]).

National and subnational governmental policies and programmes can also pose the biggest obstacles to changes in business-as-usual agricultural frontier expansion, given the powerful economic interests involved and the rent-seeking opportunities that emerge in any significant policy reform initiative [41,51]. The overarching challenge is to *align* forest maintaining policies and programmes (and the key ministries and civil servants responsible for implementation of such policies and programmes) with the broader set of programmes, government actors and stakeholders who are responsible for agriculture, finance and rural development and to *embed* these efforts within vertical systems of performance incentives and accountability that are emerging within REDD+ and commodity supply chains. This kind of horizontal and vertical policy coordination is a key component of the LED-R model, discussed in §5.

## 4. Market transformation for sustainable land use

Outside government and public policy frameworks, voluntary, non-governmental approaches have been developed for improving the social and environmental performance of land-based production systems. These initiatives have arisen, in part, because of the limited effectiveness of governments and public policy in protecting public interests against environmental degradation, and inadequate labour relations associated with production systems. The array of mechanisms that have been developed include the social and environmental Equator Principles adopted by many finance institutions, corporate social responsibility and, voluntary social and environmental standards. In this review, we focus on voluntary standards in their most recent manifestation: agricultural commodity roundtables.

The development of international voluntary social and environmental certification began in earnest in the 1990s with the development of the Forest Stewardship Council (FSC) and other certification systems for tropical timber production [52,53]. During its first 20 years, FSC certification has become widely recognized as a symbol of sustainable timber and pulp production. Legal compliance, a performance criterion common to all international standards, is difficult to achieve in emerging economies and young democracies, in which weak governmental institutions are often unable to implement laws and programmes across vast forest estates. This is one of many factors that may help explain why only 3 per cent of tropical timber production is certified under FSC [52,54].

Partly, in response to the persistent ‘niche market’ status of FSC and similar certification systems, a new system for developing social and environmental standards emerged that emphasizes the participation of a broader array of commodity supply chain actors from the very beginning, a focus on performance instead of techniques or practices, attention to a small group of key performance principles and a low bar of initial performance that grows more stringent over time [55]. (The agricultural commodity roundtables have not been formally described in the published literature. This description is informed by the lead author's involvement in the Round Table of Responsible Soy as a board member, and membership, through IPAM, in RSPO and Bonsucro.) Through multi-stakeholder agricultural commodity ‘roundtables’, voluntary standards are developed with the participation of a significant share of the entire supply chain, and with a focus on ‘pre-competitive’ certification (i.e. the exclusion of uncertified producers and processors from markets as opposed to post-competitive selection of certified products by well-informed, conscientious consumers who are sometimes willing to pay premiums). This emphasis on pre-competitive selection derives, in part, from the nature of the commodities themselves. Unlike timber, which is generally sold directly in the market place as a single-component commodity, soya bean, palm oil and sugar are usually one ingredient among many in retail products. This makes it more difficult to develop a workable consumer labelling approach.

Based on this pre-competitive certification approach and building on robust multi-stakeholder processes, ‘agricultural roundtables’ have now been developed for palm oil (Roundtable for Sustainable Palm Oil (RSPO), http://www.rspo.org/), sugar cane and sugar cane ethanol (Bonsucro, http://www.bonsucro.com), soya beans (Round Table for Responsible Soy (RTRS), http://www.responsiblesoy.org/), cotton (Better Cotton Initiative (BCI), http://bettercotton.org/) and biofuels of all types (Roundtable for Sustainable Biofuels (RSB), http://rsb.org/). A new roundtable for beef was also recently launched (Global Roundtable for Sustainable Beef, http://grsbeef.org/).

The rate of implementation in some of these agricultural commodity roundtable certification systems has been much more rapid than FSC. During its first 5 years of implementation, 14 per cent of world production of palm oil has become certified under RSPO. Bonsucro has certified 2 per cent after 3 years, and RTRS has certified 1 per cent after 2 years.^1^ These international standards place restrictions on the clearing of primary forests, and are therefore compatible with REDD+ programmes (table 2).

**Table 2.** Comparison of nine performance parameters for five REDD+ social and environmental safeguards and three commodity roundtable standards. Each safeguard or criterion is assessed with respect to the extent to which clear and detailed guidance is provided. Solid circles mean that there are extensive and/or restrictive guidelines. Half-filled circles mean that there are moderately restrictive guidelines. Open circles indicate no or little guidance or requirements. More detailed information is available through a preliminary report, ‘Global rules for sustainable farming’ and online data summary at http://www.ipam.org.br/ipam/social-and-environmental-safeguards-redd-and-commodity-roundtables. FPIC stands for free, prior and informed consent.

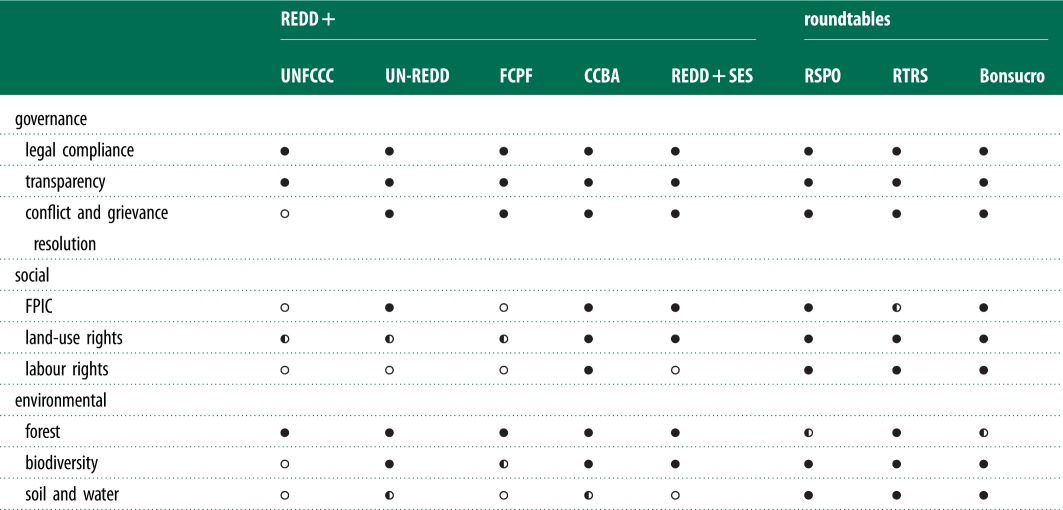

Other related processes have also resulted in voluntary commitments to make various supply chains more sustainable. These processes, like the commodity roundtables, are driven in part by the reputational risks perceived by commodity-buying companies in being associated with rainforest destruction or poor labour practices [55]. The 400-member Consumer Goods Forum (CGF), representing more than $2 trillion in annual revenues, announced their commitment to buy only beef, soya bean, palm oil, timber and pulp/paper that is produced through ‘zero net deforestation’ systems by 2020 [56]. Successive campaigns by Greenpeace to expose the association between McDonald's chicken products and deforestation-driving Amazon soya bean production and between Amazon meat packing plants and deforestation, led to moratoria with broad participation (and leadership) from soya bean industries and meat-packing companies, respectively [57]. These moratoria established forest conversion cut-off dates after which forest clearing for soya bean or beef would disqualify those products from purchase by participating buyers.

## 5. Potential synergies between REDD+ and market transformation

### (a) Lessons from REDD+

Since 2005, REDD+ has emerged as the most important international policy initiative to protect tropical forests at scale [58]. It has captured the attention of tropical forest countries, donor governments, large NGOs and multi-lateral organizations, providing an important opening for efforts to integrate tropical deforestation and land-based emissions into international climate policy. Political leaders have to varying degrees embraced REDD+ as part of a new approach to tropical forest conservation (often at significant political risk) and in some jurisdictions have started to build legal and institutional frameworks for REDD+. These investments have, combined with other efforts, contributed to globally significant emissions reductions, most notably in Brazil.

However, REDD+ has also suffered from a general lack of integration into mainstream rural development and domestic policy agendas. It has too often been treated as a special project conceived by UN diplomats and international bureaucrats, with too much ambiguity regarding core elements and operational details and too much attention to technical issues. This overly narrow, largely top-down framing of REDD+ has prevented engagement with the broader context of rural development and the challenges facing political leaders seeking to attain or remain in office [41,42,51]. Compounding this has been an overly complex set of institutions and programmes aimed at providing REDD+ finance that has so far delivered only limited funds to actors on the ground. In this context, it is not surprising that one of the principal threats to REDD+ is the lack of political support in the nations, states and provinces that are trying to implement it [16,45].

Much of the experience regarding REDD+ on the ground has also been dominated by individual projects without sufficient attention to the challenges and opportunities that exist at jurisdictional scales [42]. Private investors, project developers, the voluntary carbon market standards organizations and large international NGOs have all tended to focus on project-level approaches to REDD+ given their incentives for return on investment, their experience and past history with conservation projects, and their reluctance to engage with governments. Such a focus has deflected attention away from the hard work that is required to move REDD+ to scale and tends to reinforce the general isolation of REDD+ from the political and economic realities of rural development [41,42].

This political and economic isolation of REDD+ is also apparent in the lack of direct engagement with the drivers of deforestation, manifest in the notable absence of many of the critically important land-based sectors from the REDD+ debates, and in the general lack of participation by most vulnerable, forest-dependent communities and indigenous peoples. In both cases, the failure to engage these key rural constituencies further reinforces the view that REDD+ is yet another international forest conservation scheme imposed from above [16].

### (b) Lessons from market transformation

International commodity roundtables and market transformation initiatives have operated largely in parallel to REDD+ with an approach that has deliberately avoided dependence on cumbersome UN policy processes. Roundtables were developed, instead, with a focus on multi-stakeholder engagement focused on the supply chains of individual commodities. They were launched with 25–50% of world sales of the target commodity represented among their member companies.^2^ Each roundtable has developed its own principles and criteria for defining and measuring social and environmental performance, and has supported the certification of farms and mills that achieve these criteria. Campaigns against production systems of soya bean, beef and palm oil that are associated with tropical deforestation led by Greenpeace (and with surprising levels of cooperation and leadership by industry) have served to strengthen the perception of reputational risk among commodity traders and processors, and the retailers that they sell to, associated with buying from supply chains that are engaged in deforestation. The CGF commitment to zero net deforestation supply chains by 2020 grows out of this broader perception of risk.

Commodity roundtables have encountered important impediments that could ultimately prevent them from achieving market transformation. Performance is measured at the level of individual farms and mills, and certification can therefore be very expensive, favouring large-scale producers who are already close to meeting the standard's requirements [59]. Price premiums for certified products are often low, and many producers have therefore not been compensated for the costs of complying with and auditing farm-level performance criteria. Many of the companies that made pledges to buy certified commodities from some of the roundtables have been slow to implement those pledges, resulting in over-supply of certified production. With high costs of certification and low premiums, producers in nations with ambitious, complex land-use legislation, such as Brazil and Indonesia, are at a particular disadvantage [60].

Farmers and livestock producers have expressed frustration with demands that they receive from both the market and policy processes. Neither REDD+ nor market transformation initiatives have delivered positive incentives or technical assistance to make the changes in their production systems that are being demanded (D. Nepstad & A. Azevedo 2012, unpublished data; interviews conducted with ACRIMAT, Aliança da Terra and Aprosoja, Mato Grosso, in August 2012).

### (c) ‘Bottom-up’ convergence between REDD+ and market transformation

REDD+ has been constrained by its dependence on a cumbersome international policy process that has not delivered financing at sufficient scale, its slow progress in penetrating the rural development processes in tropical nations, and the low level of engagement among farm sectors and forest-dependent communities. Market transformation has been slowed by the lack of positive financial incentives for farmers and companies to meet social and environmental criteria of roundtables, high transaction costs and barriers to scale embedded in the farm-level certification approach, and the possible weakening of market demand for certified products. We highlight here three opportunities for linking REDD+ and market transformation to achieve potential synergies that might help to support the transition to LED-R by overcoming these constraints.

**Figure 2.**
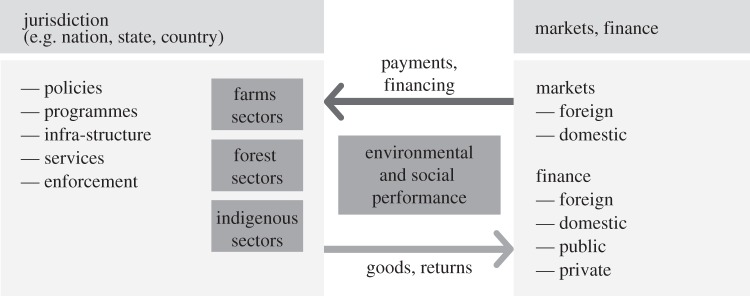
Diagram illustrating an emerging framework for linking (i) jurisdictions that are shifting to a low-emission rural development (LED-R) model with (ii) markets (e.g. agricultural commodities, but also domestic food, food, fuel and feed markets) and (iii) finance (both international and domestic, public and private). Clear, broadly accepted definitions of environmental and social performance of land-use systems that are monitored across entire jurisdictions could help to link these three spheres together.

The first opportunity is the growing potential for achieving a broadly adopted, incremental definition of social and environmental performance for LED-R that combines elements from jurisdictional REDD+ with roundtable performance standards and that could be monitored at the scale of entire jurisdictions (figure 2). This definition is needed to help link together what are currently a very fragmented set of initiatives designed to encourage transitions to more sustainable forms of land-based production that are less dependent on deforestation. Performance indicators that can be monitored at the level of jurisdictions (through satellite imagery, rural censuses or other approaches) are needed to surmount the high transaction costs of property-by-property certification in the case of roundtables and project-by-project interventions in the case of REDD+. Jurisdiction-level performance is more easily aligned with policies and governmental programmes, and is the necessary level of intervention for achieving scale in the transition to LED-R. The reduction in deforestation is a performance criterion that is particularly well suited to such a definition. REDD+ programmes measure performance against emissions reference levels developed for entire jurisdictions [37,38], whereas roundtables, moratoria and the CGF use deforestation cut-off dates and a range of definitions of the forests to which these cut-off dates apply. Convergence around the jurisdictional deforestation reference level approach to defining and measuring progress in reducing or ending deforestation might be sought as an initial step, for example, adding additional environmental and social indicators incrementally as effective mechanisms for monitoring and measuring them are developed. Jurisdictional performance could expand to include agricultural GHG emissions, for example, as has been developed for counties in the USA [61].

The second opportunity is the development of domestic programmes for delivering positive financial and technical incentives to support farm and farm community transitions to sustainable production systems that provide entry points for international finance (figure 2). Domestic financial incentives could come in the form of tax reform, sustainable farming bonds, agricultural loan programmes with interest rates that are differentiated to favour high-performing farmers, or other mechanisms [62]. Technical support in the form of rural extension services could provide the necessary knowledge transfer to farmers that are making the transition to improved production systems. These programmes could be designed to reward successful performance measured at the jurisdictional level with additional advantages flowing to individual farms and farm communities that are elevating their social and environmental performance, for example, through certification under a roundtable standard. Important precedents, such as the programme for eradicating foot-and-mouth disease and the State of Pará's performance-based incentive system for lowering county-level deforestation (‘Municípios Verdes’), demonstrate the potential of jurisdictional approaches to foster changes in landholder behaviour at scale^3^, often reinforced by peer-to-peer enforcement [63]. These domestic programmes could be strengthened through linkages to emerging performance-based international finance, such as Norway's REDD+ programme, Germany's REDD Early Mover programme, California's cap-and-trade system REDD offset programme that is under debate, or a broad range of public and private finance that is aligned with sustainable production and emissions reductions (table 1).

The third opportunity is procedural. REDD+ has been constrained by an inadequate level of participation of farm sectors [16,42] and rural communities; commodity roundtables have achieved farm sector engagement, but have failed to develop mechanisms for covering the costs of certification. These deficiencies are particularly important in the very dynamic agricultural frontiers where crop expansion into forests is proceeding most rapidly, such as the Brazilian Amazon and Indonesia, and where powerful industry elites can block the progress of both REDD+ and market transformation [41,51]. Multiple-stakeholder dialogues that develop regional definitions for environmental and social performance while providing input to the design of financial instruments to support improvements in this performance could help to engage the farm sectors and support their transition to sustainable practices. Participatory regional planning processes have succeeded in influencing policies, planning and programmes in forest frontiers [64–66]; multiple-stakeholder processes are central to the success thus far of the commodity roundtables. In this context, the GCF, in which representatives from state/provincial governments, civil society and the private sector have been collaborating for 5 years, represents one possible forum for facilitating such dialogues and taking them to scale across several jurisdictions and nations [46].

To increase the likelihood of success, these three opportunities could be pursued together, beginning in critical regions of ongoing agricultural expansion into forests. Finance institutions (public and private), farm sector organizations, civil society, government institutions and other stakeholders might become engaged in multi-sector dialogues if they are framed to include topics of the greatest relevance to regional constituencies, such as increasing production, efficiency and profitability of crop and livestock production systems, higher food security, job creation, market access, improved air quality (e.g. less vegetation burning), improved water quality and other issues [16]. The transition to sustainable production systems, including a reduction in deforestation, may have the highest chance of success in overcoming powerful vested interests in business-as-usual frontier expansion if there is a broad and growing base of political support for an alternative pathway of LED-R.

These ‘bottom-up’ processes could become mutually reinforcing if they achieve inter-regional convergence. Several mechanisms could help to facilitate this convergence. Agricultural roundtables could consider adopting jurisdictional principles and criteria that draw upon the lessons of regional multiple stakeholder processes, and that feed into these processes. The CGF could join the regional processes at strategic moments to align its strategy for achieving its 2020 zero net deforestation targets. The GCF could help to strengthen and interconnect these multiple-stakeholder processes, as could Norway, the UK, Germany and other nations with strong forest programmes. Ultimately, the lessons and agreements taking place at the regional level could help to inform and strengthen the ongoing negotiations of REDD+ and land use within the UNFCCC and associated processes.

**Figure 3.**
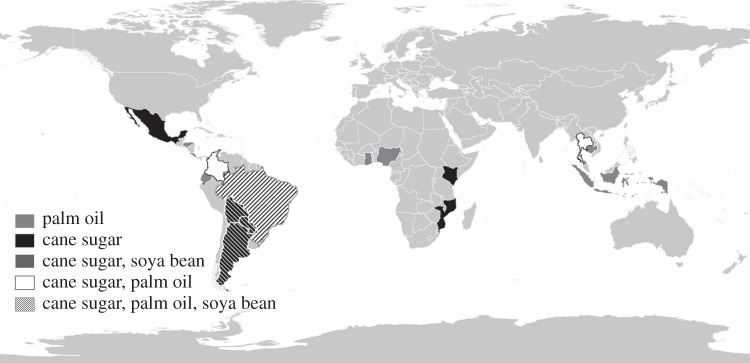
Map of nations that are developing REDD+ programmes through one of the UNFCCC affiliated programmes (participating in one of the programmes summarized in table 3) or that have states that are participating in the Governors' Climate and Forests Task Force, and that also have farm sectors that are moving into compliance with one of the agricultural commodity roundtable standards (Bonsucro, RSPO or RTRS). Data are from the sources cited in table 2 (for REDD+ programmes) and from analysis of the membership databases of the roundtables (Roundtable for Sustainable Palm Oil (http://www.rspo.org/); Bonsucro (http://www.bonsucro.com), Round Table for Responsible Soy (http://www.responsiblesoy.org/).

The convergence of REDD+, market transformation and domestic policies and programmes could begin in the nations and states where REDD+ programmes are under development and where agricultural commodity producers are already coming into compliance with one of the main standards (Bonsucro, RSPO or RTRS; figure 3). This confluence is found in 19 tropical nations. The transition to LED-R could be implemented in tropical nations with little commodity-driven deforestation as well, with a focus on domestic market transformation.

## 6. The case of Mato Grosso, Brazil

**Figure 4.**
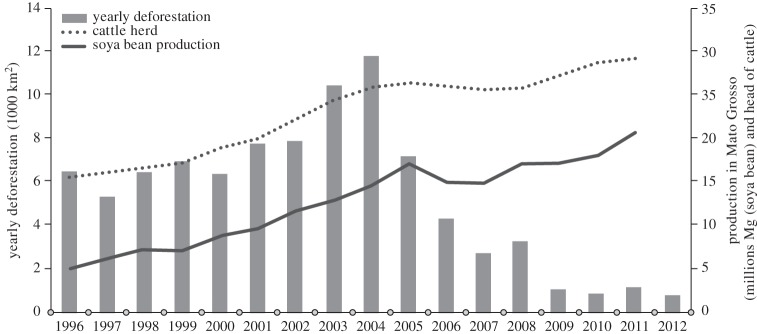
Historical deforestation, cattle herd (number of head) and soya bean production in Mato Grosso State, southeastern Amazon, Brazil [47,67].

The promise and challenge of how REDD+ and market transformation might converge is best understood in the context of actual transitions that are underway. We analyse, here, the case of Mato Grosso (MT), a 900 000 km^2^ state located in the southeastern Amazon region. It is Brazil's largest agricultural producer and was responsible for 40 per cent of the deforestation in the Brazilian Amazon region for the 10-year period ending in 2005, declining 90 per cent below its 10-year average by 2012 (figure 4) [67,68]. This is equivalent to a 0.8 per cent decline in global anthropogenic carbon dioxide emissions to the atmosphere.

The causes of the dramatic decline in MT deforestation are not fully understood, in part, because of the diverse array of policy and market interventions that unfolded in the state during the administration of Governor Blairo Maggi that ended in 2009, and that have been partially reviewed elsewhere [57,69,70]. Campaigns designed and launched by Greenpeace (described in §§4 and 5*b*) contributed to the decline [71,72]. These campaigns resulted in the soya bean moratorium of 2006 and the beef moratorium of 2009, with broad participation of civil society and industry [57]. The federal government launched its *municípios críticos* programme, restricting access to credit for farmers in high-deforestation counties [70]. The prospect of REDD+ may have created an expectation of remuneration of standing forests on private properties, inhibiting deforestation. Agricultural roundtables were also under development with strong participation from MT producers and with restrictions on deforestation.

The decline in deforestation was more palatable to the state's agriculture and livestock sectors because it did not severely impede their growth (figure 4). Beef and soya bean production continued to increase during the period, made possible by increases in stocking densities and yields of beef production systems. Cattle fattening operations have become more productive and efficient through the increased use of silage and ration derived from the expanding soya bean, maize, millet and cotton crops, especially during the dry season, and through improved breeding and pasture management [67]. With cattle pasture occupying 26 per cent of the state territory and crops only 6 per cent, small increases in cattle productivity are allowing beef production to expand on a declining area of pasture, opening pastureland for crop expansion.

These steps towards LED-R in MT are fragile at many levels. First, rising commodity prices have increased the profitability of forest conversion to soya bean and maize, replicating the circumstances in 2004 and 2005, when exchange rates favoured Mato Grosso soya bean [73]. As the amount of cattle pasture that is available for conversion to mechanized crop production diminishes, pressure will grow to convert forests. Second, there are few positive incentives for key state and municipal government actors and the farm sector to invest in building frameworks for LED-R, and a correspondingly low level of engagement in REDD+ [47]. The soya bean and beef industries, meanwhile, have lowered expectations that REDD+ will deliver benefits if they continue to support the state's decline in deforestation (D. Nepstad & A. Azevedo 2012, unpublished data. Interviews conducted with ACRIMAT, Aliança da Terra, and Aprosoja, Mato Grosso, in August 2012). The most powerful organization representing the MT soya bean sector, Aprosoja, backed away from the RTRS when that process failed to develop effective mechanisms for compensating farmers for the cost of complying with the Forest Code [60].

MT farmers and ranchers have come to view deforestation as risky [74], but are frustrated by the lack of positive incentives available to them as some forego the profits associated with legal forest conversion to crops or livestock and as they invest in improvements in their production systems to comply with roundtable standards. There are 10 different programmes that are related to deforestation in some way, each with its own definition of deforestation and none providing positive incentives for the transition to sustainability [74]. The ‘low carbon agriculture’ credit programme of US$1.5 billion per year has reached few MT farmers because of the complexity of the application process and high interest rate (5.5% per year) relative to some other agricultural credit programmes [75].

MT's further progress towards LED-R may depend upon a reframing of REDD+ to move beyond the unmet expectations for a grand global mechanism that delivers large flows of revenue into the state. The state's new REDD+ law (http://www.gcftaskforce-database.org/ReddImplementation/MatoGrosso) establishes a legal framework for programmes that could eventually reinforce the transition of farm and livestock sectors to low deforestation and higher yielding production systems as it strengthens law enforcement and technical support, attracting performance-based finance from international public and private sources. The new Brazilian Forest Code also has provisions for potential new programmes for delivering positive incentives to farmers who comply with the Code [76]. A recently launched multiple-stakeholder dialogue of farm sectors, financial institutions, government and civil society [74] identified several points of convergence on the issue of agricultural expansion and forest maintenance and restoration, and the financial instruments that could help drive further gains in yields and reductions in deforestation, that could become the seed for securing and deepening MT's transition to LED-R.

## 7. Conclusion

Preventing a vicious cycle between worsening climate change and a deepening land crisis in a world of seven billion people and rising will require, among other things, intensification of agriculture and other land-based production systems on a scale that can meet growing demand for food, fuel, feed and fibre while significantly *reducing* the current contribution of deforestation and land use to global GHG emissions while protecting and improving the rights and livelihoods of indigenous and traditional communities. For this rural development agenda to succeed, political support across a broad set of constituencies may be necessary that can neutralize or redirect powerful elites who control rural agricultural frontier dynamics in many nations while creating in-country incentives to build the institutional capacity that a LED-R model will require. A strategy for achieving synergy between REDD+ and market transformation might help to reinforce the development of this agenda in the Brazilian Amazon region and, perhaps, other critical regions of crop or livestock expansion into forests. ‘Bottom-up’ regional strategies that succeed in merging market forces, domestic policies and finance, and international finance could help inform international climate and forest policy and strengthen the market transformation to exclude unsustainable commodity producers from supply chains. In a world where climate policy is apt to remain deeply fragmented for the foreseeable future and where the pressures on land-based production are accelerating, identifying and encouraging these bottom-up policy innovations and linking them with broader structures of support and accountability offers an important path forward.

## Endnotes

1 Sources: commodity roundtable websites accessed February 20, 2013. Roundtable for Sustainable Palm Oil, http://www.rspo.org; Bonsucro, http://www.bonsucro.com; Round Table for Responsible Soy, http://www.responsiblesoy.org.

2 Data from interviews conducted by D. Nepstad in 2010 of the Directors of RSPO, RTRS and Bonsucro, based upon estimates of the aggregate purchase of each commodity by member companies expressed as a percentage of global annual production of that commodity.

3 Foot-and-mouth disease eradication programs restrict market access for cattle producers located in geographical zones that have experienced recent outbreaks of the disease, fostering powerful ‘peer-to-peer’ enforcement among ranchers (http://www.oie.int/eng/A_FMD2012/docs/en_chapitre_1.8.5.pdf, http://www.oie.int/doc/ged/D11786.PDF).
